# CDC Grand Rounds: Reducing the Burden of HPV-Associated Cancer and Disease

**Published:** 2014-01-31

**Authors:** Eileen F. Dunne, Lauri E. Markowitz, Mona Saraiya, Shannon Stokley, Amy Middleman, Elizabeth R. Unger, Alcia Williams, John Iskander

**Affiliations:** 1 National Center for HIV/AIDS, Viral Hepatitis, STD, and TB Prevention; 2 National Center for Chronic Disease Prevention and Health Promotion; 3 National Center for Immunization and Respiratory Diseases; 4 University of Oklahoma Health Sciences Center; 5 National Center for Emerging and Zoonotic Infectious Diseases; 6 Office of the Associate Director for Science, CDC

## Magnitude of the Problem

Human papillomavirus (HPV) infection is the most common sexually transmitted infection in men and women in the United States. Most sexually active persons will acquire HPV in their lifetime. Recent data indicate that approximately 79 million persons are currently infected with HPV, and 14 million persons are newly infected each year in the United States (1).

Of the more than 150 different types of HPV, approximately 40 are transmitted through sexual contact and infect the anogenital region and other mucosal sites of the body. Mucosal HPV types are classified as either high-risk HPV (oncogenic) (e.g., types 16 and 18) or low-risk HPV (e.g., types 6 and 11). High-risk HPV causes many cancers of the cervix, vagina, vulva, penis, and anus. HPV16 is linked to many oropharyngeal cancers. Low-risk HPV causes anogenital warts and recurrent respiratory papillomatosis, a rare but important condition in which warts grow in the throat and airway. Most infections cause no symptoms and are not clinically significant, but persistent infection can lead to disease or cancer.

Recent U.S. population-based studies conducted by CDC show that 66% of cervical cancers, 55% of vaginal cancers, 79% of anal cancers, and 62% of oropharyngeal cancers are attributable to HPV types 16 or 18. Each year in the United States, an estimated 26,000 new cancers are attributable to HPV, about 17,000 in women and 9,000 in men (2).

Disparities exist in HPV-associated cervical cancer rates by race/ethnicity, with higher incidence rates among Hispanic, black, and American Indian/Alaskan Native women than among whites. HPV-associated vaginal cancers are slightly more frequent among blacks, and vulvar cancers are more frequent among whites. HPV-associated oropharyngeal cancers have been increasing in frequency among both sexes, more among males than females, as well as among most racial/ethnic groups, with the exception of blacks (3). HPV-associated anal cancers have increased among males and females across all racial/ethnic groups (3).

## Evidence-Based HPV Prevention

Two HPV vaccines (bivalent and quadrivalent) are licensed by the Food and Drug Administration (FDA). Both vaccines are directed against HPV16 and HPV18, types that cause cervical cancers and other HPV-associated cancers. Quadrivalent vaccine is also directed against HPV6 and HPV11, types that cause anogenital warts. Data from clinical trials show that both vaccines, when given as a 3-dose series, have very high efficacy for prevention of vaccine type–associated cervical precancers (4–6) (Table). Quadrivalent HPV vaccine has been shown to prevent HPV16- and HPV18-associated vaginal, vulvar, and anal precancers (7,8) and HPV6- and HPV11-associated anogenital warts (9). The vaccines are prophylactic and do not prevent progression of existing infection to disease or treat existing disease (10). No clinical trial data are currently available to demonstrate efficacy for prevention of oropharyngeal or penile cancers. However, because many of these are attributable to HPV16, the HPV vaccine is likely to offer protection against these cancers as well.

The Advisory Committee on Immunization Practices (ACIP) recommends that girls and boys be routinely vaccinated at age 11 or 12 years; vaccine may be given starting at age 9 years (11–13). In addition, for those who were not vaccinated when they were younger, all girls/young women through age 26 years (12) and all boys/young men through age 21 years should be vaccinated (13). ACIP recommends that gay, bisexual, and other men who have sex with men be vaccinated through age 26 years (13). ACIP considered data on vaccine efficacy and safety, disease burden attributable to HPV, cost-effectiveness of vaccination, and programmatic issues to develop recommendations.

The HPV vaccine is covered by most private health insurance and government insurance programs. For uninsured, Medicaid-eligible children of American Indian/Alaska Native descent and underinsured persons aged ≤18 years, the Vaccines for Children Program (VFC) provides federally purchased vaccines recommended by ACIP at no cost to those eligible. Approximately 39% of adolescents aged 13–17 years are eligible for VFC vaccines; nationally, approximately 44,000 vaccination provider sites are enrolled in the VFC program (14). Most vaccine being used in the United States is quadrivalent HPV vaccine.

## Current and Future Challenges for HPV Prevention

Improving vaccination coverage is important to reduce the burden of cancer and disease caused by HPV. National HPV vaccination coverage data reveal a concerning trend among female adolescents aged 13–17 years. In comparison with other vaccinations recommended for adolescents (e.g., tetanus, diphtheria, and acellular pertussis vaccine [Tdap] and meningococcal conjugate vaccine [MenACWY]), HPV vaccination coverage for adolescent girls has increased slowly and remains far below *Healthy People 2020* targets; an average increase in HPV vaccination coverage of 6 percentage points was observed each year from 2007 through 2011, but no increase occurred from 2011 to 2012 (15). Coverage for adolescent girls with at least 1 dose of HPV vaccine was 53.8%, and coverage with all 3 doses was 33.4% in 2012 (15) (Figure). Wide variations by state also were observed in 2012, with coverage of 3 doses among adolescent girls ranging from a low of 12.1% in Mississippi to a high of 57.7% in Rhode Island (15).

Strategies to increase adolescent HPV vaccination coverage rates in the United States include reminder/recall systems to increase first dose and series completion rates; standing orders for vaccination; education of patients, parents, and health-care providers; health insurance reforms to reduce out-of-pocket costs for vaccines; and increasing the use of alternative vaccination sites (e.g., schools). School requirements have been found to increase vaccination coverage for Tdap and MenACWY; however, HPV vaccine is required for school entry in only a few jurisdictions. Health-care providers should administer HPV vaccine during visits when Tdap and MenACWY are administered. The single most important predictor of vaccination in the clinical setting is a strong recommendation from a health-care provider (16).

Monitoring for adverse events after HPV vaccination is occurring through several systems. Two federal systems are the Vaccine Adverse Event Reporting System (VAERS) and the Vaccine Safety Datalink (VSD). From June 2006 through March 2013, approximately 56 million doses of HPV4 were distributed in the United States. During that period, VAERS received a total of 21,194 reports of adverse events occurring in females after receipt of HPV4; 92.1% of these events were classified as nonserious (17). Among nonserious adverse events, the most commonly reported generalized symptoms were syncope (fainting), dizziness, nausea, headache, fever, and urticaria (hives); the most commonly reported local symptoms were injection-site pain, redness, and swelling (17). Among 600,588 doses of HPV4 administered to females aged 9–26 years in VSD, no significant increased risk was observed for any of the prespecified adverse events after vaccination, including Guillain-Barré syndrome, seizures, syncope, appendicitis, stroke, venous thromboembolism, or anaphylaxis and other allergic reactions (18).

Evaluations to assess the impact of HPV vaccine on biologic outcomes (e.g., HPV prevalence in the population, incidence of anogenital warts and HPV-associated precancers/cancers, and HPV type distribution in lesions) are currently underway but are associated with challenges. For example, most HPV-associated disease outcomes are not reported nationally and therefore require new data collection systems. Also, changes in cervical cancer screening recommendations and in terminology used for pathology will impact surveillance for cervical precancers. Moreover, the impact on HPV-associated cancers requires a decade or longer to measure. Data being used to measure biologic impact include those from the National Health and Nutrition Examination Survey (NHANES), sentinel surveillance systems, cancer registries, administrative data such as Marketscan and Medicaid data, and other special evaluations. Ongoing impact monitoring and surveillance of HPV vaccination are important to assess the duration of vaccine-induced protection, the potential replacement of vaccine HPV types with nonvaccine types, and the efficacy of <3 vaccine doses. Despite challenges in measuring vaccination impact, recent NHANES data demonstrate reductions of the prevalence of HPV types 6, 11, 16, and 18 (19), and Marketscan data indicate a reduction of the prevalence of anogenital warts (20).

## Conclusion

The burden and cost of HPV-associated disease and cancer remain an important public health problem. Reducing the burden of HPV-associated cancer and disease through vaccination requires an integrated approach that includes clinical medicine, public health, and public policy. Two FDA-licensed prophylactic HPV vaccines are safe, well tolerated, and highly effective. Vaccination is routinely recommended for girls and boys aged 11 or 12 years; however, vaccination coverage is well below *Healthy People 2020* targets. An important public health goal is enhancing HPV disease prevention by improving vaccination coverage through public policy and clinical practice. Programs are in place to monitor coverage, safety, and postlicensure impact of HPV vaccine in the United States, and these will continue to provide important information on the HPV vaccination program.

## Figures and Tables

**FIGURE:**
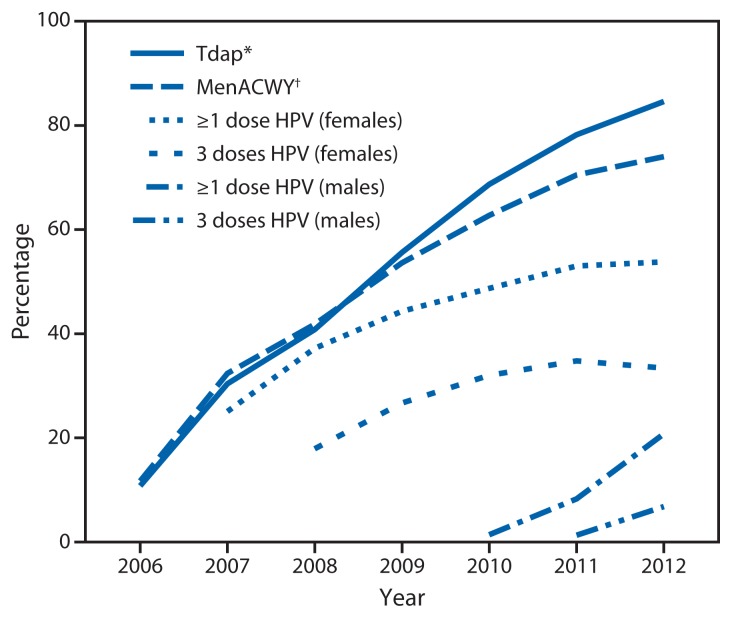
Estimated vaccination coverage with selected vaccines and doses among adolescents aged 13–17 years, by survey year — National Immunization Survey–Teen, United States, 2006–2012 **Abbreviations:** Tdap = tetanus toxoid, reduced diphtheria toxoid, and acellular pertussis; MenACWY = meningococcal conjugate; HPV = human papillomavirus. **Source:** CDC. National and state vaccination coverage among adolescents aged 13–17 years—United States, 2012. MMWR 2013;62:685–93. Available at http://www.cdc.gov/mmwr/preview/mmwrhtml/mm6234a1.htm. ^*^≥1 dose Tdap on or after age 10 years. ^†^≥1 dose MenACWY.

**TABLE:** Results of selected clinical trials* on human papillomavirus (HPV) vaccine efficacy against HPV vaccine-type precancers and anogenital warts

Outcome	Vaccine	Sex	Vaccine efficacy
Cervical precancer	Bivalent and quadrivalent	Females	>93%
Vaginal/Vulvar precancer	Quadrivalent	Females	100%
Anal precancer	Quadrivalent	Males	75%
Anogenital warts	Quadrivalent	Females	99%
		Males	89%

**Sources:** Paavonen J, Naud P, Salmeron J, et al. Efficacy of human papillomavirus (HPV)-16/18 AS04-adjuvanted vaccine against cervical infection and precancer caused by oncogenic HPV types (PATRICIA): final analysis of a double-blind, randomised study in young women. Lancet 2009;374:301–14. Kjaer SK, Sigurdsson K, Iversen OE, et al. A pooled analysis of continued prophylactic efficacy of quadrivalent human papillomavirus (types 6/11/16/18) vaccine against high-grade cervical and external genital lesions. Cancer Prev Res 2009;2:868–78. Future I/II Study Group. Four year efficacy of prophylactic human papillomavirus quadrivalent vaccine against low grade cervical, vulvar, and vaginal intraepithelial neoplasia and anogenital warts: randomised controlled trial. BMJ 2010;341:c3493. Guiliano AR, Palefsky JM, Goldstone S, Moreira ED, et al. Efficacy of quadrivalent HPV vaccine against HPV infection and disease in males. N Engl J Med 2011;364:401–11. Palefsky J, Giuliano AR, Goldstone S, et al. HPV vaccine against anal HPV infection and anal intraepithelial neoplasia. N Engl J Med 2011;365:1576–85. Lehtinen M, Paavonen J, Wheeler CM, et al. Overall efficacy of HPV-16/18 AS04-adjuvanted vaccine against grade 3 or greater cervical intraepithelial neoplasia: 4-year end-of-study analysis of the randomised, double-blind PATRICIA trial. Lancet Oncol 2012;13:89–99.

* Population includes the per-protocol and according-to-protocol population. Subjects received all 3 doses, and cases were counted 1 month after dose 3.
